# Stress Buildup Upon Crystallization of GeTe Thin Films: Curvature Measurements and Modelling

**DOI:** 10.3390/nano10061247

**Published:** 2020-06-26

**Authors:** Rajkiran Tholapi, Manon Gallard, Nelly Burle, Christophe Guichet, Stephanie Escoubas, Magali Putero, Cristian Mocuta, Marie-Ingrid Richard, Rebecca Chahine, Chiara Sabbione, Mathieu Bernard, Leila Fellouh, Pierre Noé, Olivier Thomas

**Affiliations:** 1 Aix Marseille Univ, U. Toulon, CNRS, IM2NP (Institut Matériaux Microélectronique et Nanosciences de Provence), Campus St-Jérôme, 13397 Marseille CEDEX 20, France; manon.gallard@im2np.fr (M.G.); nelly.burle@im2np.fr (N.B.); christophe.guichet@im2np.fr (C.G.); stephanie.escoubas@im2np.fr (S.E.); magali.putero@im2np.fr (M.P.); mrichard@esrf.fr (M.-I.R.); 2 Synchrotron SOLEIL, l’Orme des Merisiers, Saint-Aubin–BP 48, 91192 Gif-sur-Yvette, France; cristian.mocuta@synchrotron-soleil.fr; 3 ESRF, The European Synchrotron, ID01 Beamline, 71 Rue des Martyrs, 38043 Grenoble, France; 4 University Grenoble Alpes, CEA (Commissariat à l’Energie Atomique et aux Énergies Alternatives), LETI (Laboratoire d’Electronique et des Technologies de l’Information), F-38000 Grenoble, France; chahinebecca@gmail.com (R.C.); chiara.sabbione@cea.fr (C.S.); mathieu.bernard@cea.fr (M.B.); leila.fellouh@cea.fr (L.F.); pierre.noe@cea.fr (P.N.)

**Keywords:** phase-change materials, wafer curvature, GeTe, stress, crystallization

## Abstract

Phase change materials are attractive materials for non-volatile memories because of their ability to switch reversibly between an amorphous and a crystal phase. The volume change upon crystallization induces mechanical stress that needs to be understood and controlled. In this work, we monitor stress evolution during crystallization in thin GeTe films capped with SiO*_x_*, using optical curvature measurements. A 150 MPa tensile stress buildup is measured when the 100 nm thick film crystallizes. Stress evolution is a result of viscosity increase with time and a tentative model is proposed that renders qualitatively the observed features.

## 1. Introduction

A number of Te-based alloys (e.g., Ge–Sb–Te alloys located on the GeTe–Sb_2_Te_3_ pseudo-binary tie line, such as GeTe, Ge_2_Sb_2_Te_5_, Ge_1_Sb_2_Te_4_ and so on) can be reversibly switched between an amorphous and a crystalline phase. This property together with a large contrast in optical reflectivity and electrical resistivity makes them very attractive as optical and/or electronic non-volatile memories. One of the most studied alloys for memory devices is Ge_2_Sb_2_Te_5._ These so-called phase-change materials have been widely investigated for many years [1,2,3,4,5] but because of their inherent complexity, many questions remain open. For example, the quantitative knowledge of the crystallization kinetics as a function of the material environment (capping layer, doping, confinement in 1, 2 or 3D) is still lacking. This information is crucial for device fabrication since the data programming speed directly relies on the phase transition. In addition, the amorphous to crystal transition is accompanied by a large increase in density (7–8% for GeTe). The resulting stresses may be responsible for inducing defects and impacting the reliability of devices, but also may modify the properties of the phase-change material. Understanding and controlling the stress buildup caused by crystallization is, thus, a major concern for phase-change materials.

In the present work, we measure the stress evolution in GeTe’s initially amorphous layers during annealing via in situ curvature measurements. From these measurements, we propose some tentative understanding of the mechanisms at work and propose the bases for modelling stress evolution during crystallization.

This particular study is focused on “thick” GeTe films (100 nm) since we have shown in a previous work [6] that the overall stress behavior is similar for thinner films. Here, we want to focus on the physical understanding of stress development and relaxation related to film crystallization.

## 2. Materials and Methods

The 100 nm thick GeTe layers were deposited at room temperature by magnetron sputtering on 100 µm thick Si (001) substrates. The thickness of the GeTe layer was assessed from the spacing of the Kiessig fringes observed in X-ray reflectivity measurements. The deposited films are slightly Ge-rich (Ge_0.52_Te_0.48_), which was confirmed by Rutherford Backscattering Spectrometry (RBS) and Wavelength Dispersive X-ray Fluorescence (WDXRF) with an error of about ±1%. As will be shown later on, the thin substrates used for deposition allowed for a good accuracy in stress-induced curvature measurements. A 10 nm SiO_2_ protective capping layer was deposited in the same sputtering chamber without breaking the vacuum in order to prevent any surface oxidation of GeTe films. Surface oxidation has been shown to profoundly modify the crystallization behavior in thin chalcogenide films [7]. It is, thus, very important to keep an oxygen-free surface during annealing. The 1 × 1cm^2^ coupons were then cut and loaded in a homemade vacuum chamber equipped with an optical viewport and a heating plate. Once the desired pressure (10^−6^ mbar) was reached, the sample curvature was monitored as a function of time or temperature using a multi-beam optical sensor (MOS) from k-Space Associates (acquisition frequency from 1 to 10 Hz).

**Figure 1:**
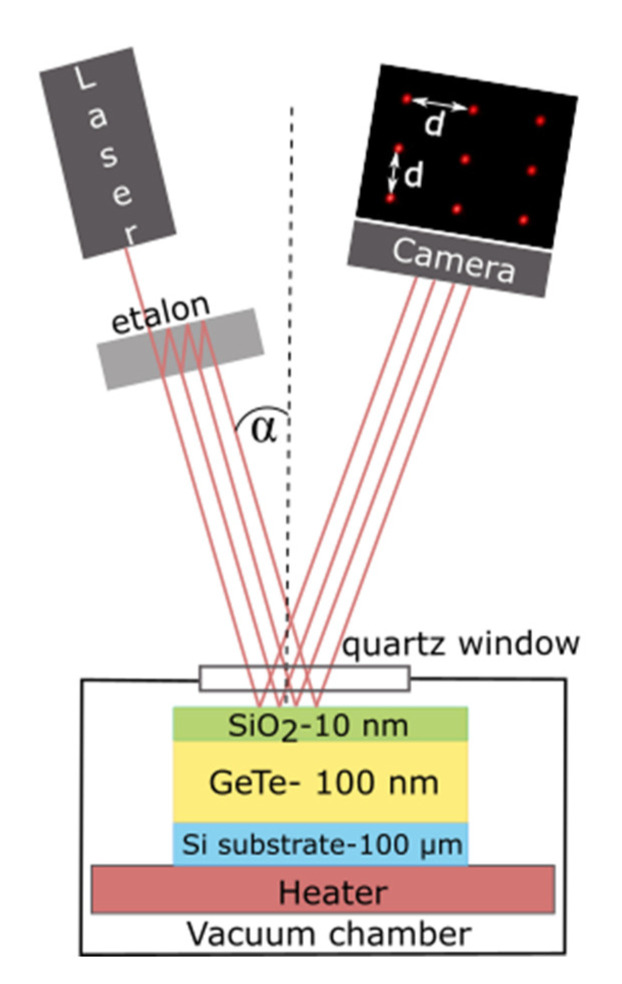
Schematic of wafer curvature measurement setup and sample layout of films used for isothermal measurements. The components in the figure are not to scale.

The curvature measurement works by monitoring a 3 × 3 matrix of reflected laser spots from the sample surface, as shown in Figure 1. The inter-spot distance (*d*) observed on the Charge- Coupled Device (CCD) camera is then compared with respect to a reference (*d_0_*) with known curvature. Later, the sample curvature is calculated by using the Equation (1), where *L* and *α* are the sample to detector (CCD camera) distance and the incident angle of the beam on the sample surface, respectively.

(1) $$F=\frac{\cos\alpha }{2L}\;\;\frac{d-d_{0}}{d_{0}}$$

The small lateral dimensions of our samples as well as the large thickness ratio between film and substrate makes Stoney’s formula valid for analysis. Hence, the force per unit length *F* applied by the coating on the substrate is given by Equation (2):

(2) $$F=\frac{M_{S}h_{S}{}^{2}}{6}\left(\kappa -\kappa_{0}\right)$$

where *M_s_* is the biaxial modulus of the substrate (180 GPa for (001) Si), *h_s_* is the thickness of the substrate (100 µm, here), *κ* is the coated substrate curvature and *κ_0_* is the bare substrate curvature.

In the present experimental approach, we will use the curvature of as-deposited samples at room temperature (RT) as *κ_0_*. This implies that the represented force (*F*) is a relative force with respect to the one arising from RT residual stresses in the deposited films (always compressive in these samples). In addition, the stress evolution can be calculated by dividing the measured force with the thickness of the active layer (100 nm of GeTe, in this case).

## 3. Results

**Figure 2:**
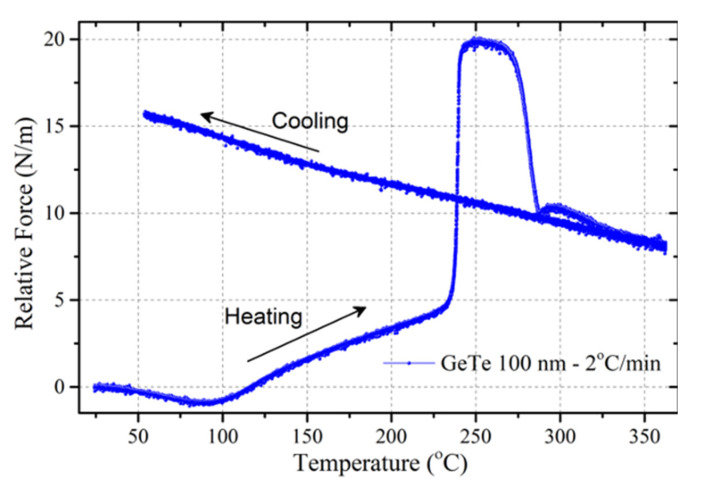
Relative force as a function of temperature for a 100 nm thick GeTe film deposited on Si and capped with 10 nm SiO_2_. Heating rate is 2 °C/min.

The evolution of the relative force as a function of annealing temperature (*T*) in a 100 nm GeTe thin film is shown in Figure 2 (heating rate is 2 °C/min). A number of characteristic features appear in this evolution.

Starting from *F* = 0 at RT, the first compressive evolution (until 90 °C) can be assigned to the thermoelastic behavior of the amorphous film. It is reversible, as seen in additional measurements (not shown here). Above ~90 °C, the force increases and shows a clear tensile evolution in the amorphous film. This can be the consequence of a densification of the film and/or of stress relaxation. At *T* = 238 °C = *T_X_*, a sharp force increase towards tension (+15 N/m) is evidenced and has been clearly associated with crystallization by in situ combined X-ray diffraction and curvature measurements [8]. Around 50 °C above *T_X_*, a steep compressive drop results from excess Ge crystallization [8]. In the cooling regime, the force exhibits a linear behavior corresponding to the thermoelastic straining of the crystallized GeTe film. X-ray diffraction measurements show that the film is non-textured with rhombohedral *R3m* structure.

**Figure 3:**
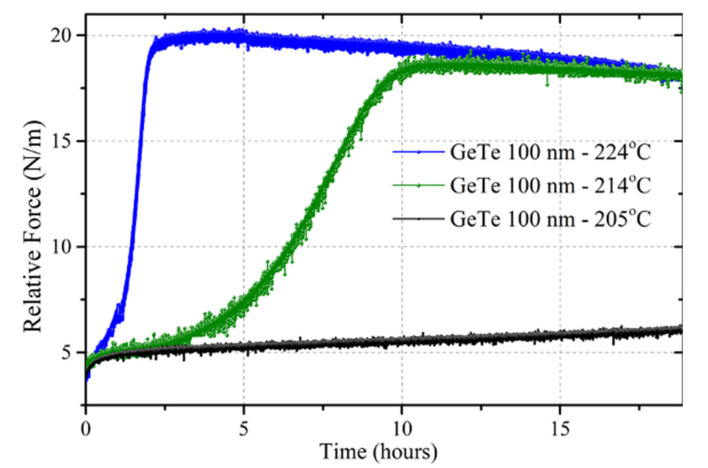
Relative force as a function of time for a 100 nm thick GeTe film deposited on Si and capped with 10 nm SiO_2_. Reported temperatures are 224, 214 and 205 °C, respectively.

Isochronal experiments, such as this one, allow for a quick investigation of the various structural and mechanical phenomena occurring as a function of temperature. On the other hand, a detailed understanding calls for isothermal measurements, since many physical parameters depend on temperature and time (growth rate, viscosity, etc.). In order to investigate more details pertaining to the stress evolution during crystallization, we have, thus, performed isothermal curvature measurements. Figure 3 shows the evolution of force during isothermal annealing at 214 and 224 °C (respectively, 24 and 14 °C below *T_X_*) for 19 h. During annealing, the film crystallizes and a tensile force increase +13 N/m at 214 °C and +15N/m at 224 °C is observed during a first regime, followed by a very slow stress relaxation in the crystalline film. The time to reach the plateau is about 2 h at 224 °C and 10 h at 214 °C. Interestingly, these numbers yield an activation energy of 3.4 eV, in perfect agreement with the activation energy for crystallization deduced from in situ X-ray diffraction experiments [9] on a 100 nm film from the same batch and in very good agreement with an activation energy of 3.14 eV, determined by differential scanning calorimetry [10] on GeTe thin films prepared by pulsed laser deposition.

A similar experiment has been performed at 205 °C and is shown in Figure 3. At this lower annealing temperature, a very slow stress relaxation is observed with no sign of crystallization even after 19 h.

## 4. Discussion

The force increase upon crystallization (at *T_X_*) translates into a stress-increase of +150 MPa. This tensile evolution is qualitatively in accordance with densification. X-ray reflectivity measurements performed in situ [8] yield a density increase of 8%, in agreement with results reported in the literature [11]. The corresponding volume change is associated with an eigenstrain (given by Equation (3)) of −2.7%.

(3) $$\varepsilon_{0}=\frac{1}{3}\frac{\Delta V}{V}$$

If one assumes a fully elastic behavior of the GeTe film, perfect adhesion at the interface as well as isotropic elastic behavior with Young’s modulus *E* and Poisson’s ratio *ν*, the amorphous to crystal transformation yields a stress increase given by Equation (4):

(4) $$\Delta \sigma =-\frac{E}{1-\nu }\varepsilon_{0}$$

*R3m* GeTe is reported to be a very anisotropic material and its elastic constants have been calculated by Shaltaf et al. [12]. Young’s modulus can vary from 48 to 142 GPa depending on crystallographic direction, the c-axis being the softest. Therefore, theoretically, in an untextured film, a stress buildup of the order of 4–5 GPa is expected upon crystallization. This is considerably larger (more than 25-times) than what is measured. In the same way, the fully elastic model predicts a change in film thickness (Equation (5)):

(5) $$\frac{\Delta h}{h}=\frac{1+\nu }{1-\nu }\varepsilon_{0}$$

of the order of 2*ε_0_* much larger than the measured value (by X-ray reflectivity) of −7.5%.

These very large discrepancies demonstrate that a very large fraction of the stress is relieved by plastic flow in the amorphous phase as was already pointed out by Pedersen et al. [13]. If one considers the crystallization process, one can assume that in the initial stages, the crystalline nuclei are surrounded by amorphous material. The stress relaxation experiment performed on fully crystallized films indicates a very weak relaxation. Therefore, in these crystallites, the stress change, which can be calculated by assuming the same elastic constants between crystal and amorphous matrix [14], as given in Equation (6), will be mostly accommodated via viscous flow in the glassy material. In such an interpretation, the viscosity of the amorphous chalcogenide is critical in determining the stress buildup.

(6) $$\Delta \sigma =-\frac{2}{3}\frac{E}{1-\nu }\varepsilon_{0}$$

More generally, the viscosity of glasses is a fundamental physical parameter [15,16]. The temperature-dependence of viscosity is a key to understand the nature of glass transition and describe the dynamics of glassy materials. It is common to describe glassy materials as strong or fragile [15], depending on whether their viscosity exhibits a simple Arrhenius behavior (such as SiO_2_, for example) or a large departure from simple Arrhenius behavior with an important viscosity increase [15] when cooling toward the glass transition. The MYEGA (Mauro Yue Ellison Gupta Allan) model [16] gives a description of viscosity as a function of temperature, including a fragility index. Concerning amorphous GeTe, a fragile behavior has been reported [17,18]. The structural relaxation in an amorphous film is governed by a relaxation time *τ*, which is simply the ratio of the viscosity by some modulus of elasticity [19,20]. It is generally considered that *τ* is in the order of 100 s [15,16] at the glass transition temperature *T_g_*. Below *T_g_*, *τ* increases very rapidly in accordance with the reported fragility of GeTe. Hence, the stress evolution in GeTe thin films upon crystallization strongly depends on the difference between the glass transition temperature *T_g_* and the crystallization temperature *T_X_*. The reported glass transition temperature for GeTe is 194 °C in nanoparticles [17] and 172 [17] or 159 °C [18] in thin films. It is important to emphasize that these numbers are obtained from uncapped samples and may reflect the influence of surface oxidation.

Stress relaxation in the amorphous GeTe film (205 °C annealed sample) cannot be described by a simple exponential behavior. This behavior has already been observed in amorphous Si, Ge or chalcogenide films [19,21] and has been associated with a time-dependent viscosity. Viscosity increases with time and this time dependence is caused by structural relaxation. Models attribute viscous flow to irreversible shear rearrangements [22] that occur at specific sites or flow defects. A unimolecular or bimolecular annihilation reaction of the flow defects yields respectively an exponential or a linear increase of viscosity as a function of time [21]. In the following, one aims at providing guidelines for modelling the isothermal stress buildup during crystallization, as measured by in situ curvature measurements. Considering the complexity of the involved physical phenomena (temperature and time-dependent viscosity and crystallization kinetics), quantitative expectations are out of reach. Interesting insights may be obtained from the model of Zhang and d’Heurle [23,24], which was originally derived to describe stress buildup during silicide formation by reactive formation between a metal film and a silicon substrate. In this model, the force evolution during the growth of the silicide layer (thickness *h*) is described as resulting from a competition between growth, with an eigenstress *σ_0_* and relaxation described by a relaxation time *τ*:

(7) $$F\left(t\right)=\int_{0}^{h\left(t\right)}\sigma_{0}\exp\left(-\frac{t-h^{-1}\left(z\right)}{\tau }\right)dz$$

**Figure 4:**
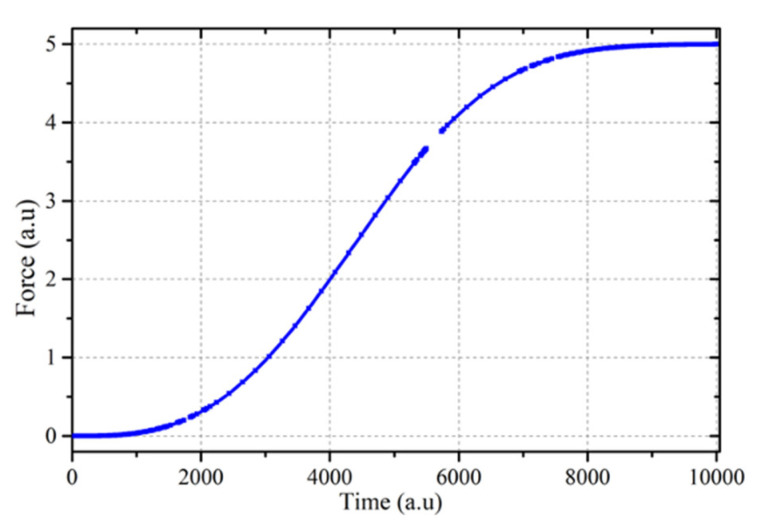
Force as a function of time for a GeTe film modelled using Equation (7) and an exponentially increasing time-dependent viscosity.

Equation (7) predicts a dumbbell behavior with an exponential relaxation at long time once there is no more unreacted metal. The maximum force reached during reaction depends on the relative growth and relaxation kinetics. The force evolution during the crystallization of a GeTe thin film (Figure 3) does not follow this behavior, since the force at long time reaches a plateau except for a very slow relaxation in the crystallized layer. The stress behavior in Figure 3 clearly shows the evolution of viscosity in the amorphous phase from a very low initial viscosity to a much higher one at long time. It is this high viscosity stage that freezes some residual stress from the densification process associated with crystallization. It is, thus, necessary to use a time-dependent relaxation time in Equation (7) in order to take into account time-dependent viscosity. In Figure 4, an exponentially increasing viscosity has been used together with JMAK (Johnson Mehl Avrami Kolmogorov) crystallization kinetics [25], using a JMAK exponent *n* equal to 3. The comparison with Figure 3 indicates that from a qualitative point of view, the main features of the force evolution have been captured.

## 5. Conclusions

In situ curvature measurements have been performed during ramp annealing on 100 nm thick GeTe films capped with 10 nm SiO_2_. Crystallization is associated with a 150 MPa stress buildup. Isothermal measurements allow the capturing of stress evolution during crystallization or—at lower temperatures—stress relaxation in the amorphous phase. The evolution of stress upon crystallization is triggered by the eigenstrain associated with volume-change together with the time-evolution of the viscosity of the amorphous phase. A tentative model is presented that captures qualitatively the observed features.

## References

[1] Raoux S. (2009). Phase Change Materials. Annu. Rev. Mater. Res..

[2] Noé P., Vallée C., Hippert F., Fillot F., Raty J.-Y. (2017). Phase-change materials for non-volatile memory devices: From technological challenges to materials science issues. Semicond. Sci. Technol..

[3] Zhang W., Mazzarello R., Wuttig M., Ma E. (2019). Designing crystallization in phase-change materials for universal memory and neuro-inspired computing. Nat. Rev. Mater..

[4] Lotnyk A., Behrens M., Rauschenbach B. (2019). Phase change thin films for non-volatile memory applications. Nanoscale Adv..

[5] Le Gallo M., Sebastian A. (2020). An overview of phase-change memory device physics. J. Phys. D Appl. Phys..

[6] Ben Yahia B., Amara M., Gallard M., Burle N., Escoubas S., Guichet C., Putero M., Mocuta C., Richard M.-I., Chahine R. (2018). In situ monitoring of stress change in GeTe thin films during thermal annealing and crystallization. Micro Nano Eng..

[7] Noe P., Sabbione C., Bernier N., Castellani N., Fillot F., Hippert F. (2016). Impact of interfaces on scenario of crystallization of phase change materials. Acta Mater..

[8] Gallard M., Amara M.S., Putero M., Burle N., Guichet C., Escoubas S., Richard M.-I., Mocuta C., Chahine R.R., Bernard M. (2020). New insights into thermomechanical behavior of GeTe thin films during crystallization. Acta Mater..

[9] Gallard M. (2019). Etude in situ de la cristallisation et des contraintes dans des nanostructures de GeTe par diffraction du rayonnement X synchrotron. Ph.D. Thesis.

[10] Sun X., Thelander E., Gerlach J.W., Decker U., Rauschenbach B. (2015). Crystallization kinetics of GeTe phase-change thin films grown by pulsed laser deposition. J. Phys. D Appl. Phys..

[11] Zhou X., Dong W., Zhang H., Simpson R. (2015). A zero density change phase change memory material: GeTe-O structural characteristics upon crystallisation. Sci. Rep..

[12] Shaltaf R., Durgun E., Raty J.-Y., Ghosez P., Gonze X. (2008). Dynamical, dielectric, and elastic properties of GeTe investigated with first-principles density functional theory. Phys. Rev. B.

[13] Pedersen T.P.L., Kalb J., Njoroge W.K., Wamwangi D., Wuttig M., Spaepen F. (2001). Mechanical stresses upon crystallization in phase change materials. Appl. Phys. Lett..

[14] Lee J.K., Barnett D.M., Aaronson H.I. (1977). The elastic strain energy of coherent ellipsoidal precipitates in anisotropic crystalline solids. Met. Mater. Trans. A.

[15] Angell C.A. (1995). Formation of Glasses from Liquids and Biopolymers. Science.

[16] Mauro J.C., Yue Y., Ellison A.J., Gupta P.K., Allan U.C. (2009). Viscosity of glass-forming liquids. Proc. Natl. Acad. Sci..

[17] Chen B., De Wal D., Brink G.H.T., Palasantzas G., Kooi B.J. (2017). Resolving crystallization kinetics of GeTe phase-change nanoparticles by ultrafast calorimetry. Cryst. Growth Des..

[18] Chen Y., Wang G., Song L., Shen X., Wang J.-Q., Huo J., Wang R., Xu T., Dai S., Nie Q. (2017). Unraveling the crystallization kinetics of supercooled liquid GeTe by ultrafast calorimetry. Cryst. Growth Des..

[19] Witvrouw A., Spaepen F. (1993). Viscosity and elastic constants of amorphous Si and Ge. J. Appl. Phys..

[20] Witvrouw A., Spaepen F. (1993). Determination of the plane stress elastic constants of thin films from substrate curvature measurements: Applications to amorphous metals. J. Appl. Phys..

[21] Kalb J., Spaepen F., Pedersen T.P.L., Wuttig M. (2003). Viscosity and elastic constants of thin films of amorphous Te alloys used for optical data storage. J. Appl. Phys..

[22] Taub A., Spaepen F. (1980). The kinetics of structural relaxation of a metallic glass. Acta Met..

[23] Zhang S.-L., D’Heurle F. (1992). Stresses from solid state reactions: A simple model, silicides. Thin Solid Films.

[24] Rivero C., Gergaud P., Gailhanou M., Boivin P., Fornara P., Niel S., Thomas O. (2005). Stress development and relaxation during reaction of a cobalt film with a silicon substrate. Defect Diffus. Forum.

[25] Christian J.W. (2002). The Theory of Transformations in Metals and Alloys.

